# Segmental Arterial Mediolysis of Omental Arteries with Haemoperitoneum: Case Report with Embolization of the Left Omental Artery and Brief Review of Literature

**DOI:** 10.1155/2018/4749356

**Published:** 2018-07-18

**Authors:** Gernot Rott, Frieder Boecker

**Affiliations:** Radiological Department, Bethesda-Hospital Duisburg, Heerstr. 219, 47053 Duisburg, Germany

## Abstract

Segmental arterial mediolysis of an omental artery is an exceptionally rare condition. A 69-year-old man presented with haemoperitoneum six days after being hospitalized due to pneumogenic sepsis. Computed tomography of the abdomen showed a short segment dilatation of an omental artery in the left upper abdomen, compatible with segmental arterial mediolysis. Angiographic examination revealed alterations of omental branches of the right gastroepiploic artery and an aneurysm of the left omental artery, both characteristic of segmental arterial mediolysis. Embolization of the left omental artery with use of N-butyl-2-cyanoacrylate was performed. The postinterventional course was uneventful with increase of haemoglobin levels and without symptoms of omental infarction. Transcatheter embolization in the setting of haemoperitoneum due to segmental arterial mediolysis of an omental branch is technically feasible and a valuable alternative to emergency operation.

## 1. Introduction

Segmental arterial mediolysis (SAM) is an uncommon, nonatherosclerotic, noninflammatory arteriopathy of unknown pathogenesis characterized by lysis of the outer arterial media resulting in dissecting hematomas and/or dissecting aneurysms of primarily medium- to large-sized splanchnic arteries. The diagnosis of SAM historically has been confirmed by histopathologic examination of the arteries involved; in modern practice, however, it is diagnosed with imaging [1–4]. The diagnosis is made radiographically by demonstrating the typical radiographic features of focal aneurysms, beading, and narrowing of affected splanchnic and renal vessels, in patients without clinical and laboratory findings indicating a vasculitis or fibromuscular dysplasia as primary diagnosis.

Recently SAM has been of interest particularly in radiological circles as the potential for investigating radiological therapeutic modalities. In the acute stage of SAM complicated by haemorrhage related to ruptured aneurysms, interventional treatment with transcatheter embolization or placement of covered stents can be the treatment of first choice. Most publications on the subject are case reports and small case series of embolization of large or middle calibre splanchnic arteries, respectively, the splenic, hepatic, gastroduodenal, gastroepiploic, or pancreaticoduodenal arteries [2, 5].

Very few cases of embolization of omental arteries due to bleeding have been published, however almost exclusively in cases of so-called idiopathic rupture of omental arteries.

To our knowledge, this is the first description of a successful embolization of an omental artery (omental branch) in the setting of acute bleeding attributable to SAM.

## 2. Case Report

A 69-year-old man presented with dyspnoea, relapsing dizziness, falls, and systemic inflammatory response syndrome in our institution. Notable lab values were a white blood count of 17.7 /*μ*l (normal range 4.0-10.0 /*μ*l), an elevated C-reactive protein of 9.6 ng/ml (normal < 0.5 ng/ml), a haemoglobin level of 13.5 g/dl (normal range 13.5-17.5 g/dl), and an elevated international normalized ratio (INR) of 1.41 without anticoagulant medication.

**Figure 1:**
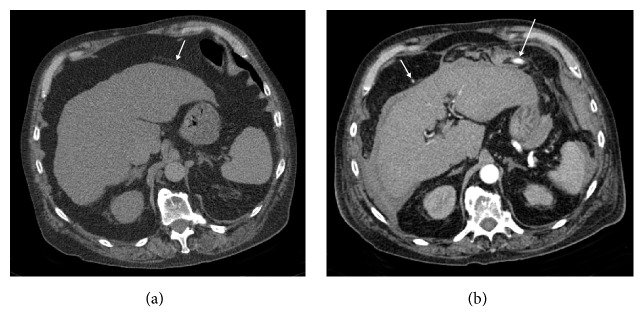
Computed tomography (CT) images. CT on admission (a) demonstrating liver cirrhosis with the greater omentum in the upper abdomen and tiny omental vessels (arrow) in the left upper abdomen. Arterial phase of contrast-enhanced computed tomography image six days later (b) with haemoperitoneum, segmental dilatated omental artery in the left (arrow), and spot of enhanced tiny omental artery in the right upper abdomen (small arrow).

Initial workup included computed tomography (CT) of the chest to rule out pulmonary embolism, which revealed right lower lobe pneumonia. In doing so, scans of the upper parts of the abdomen demonstrated liver cirrhosis without ascites or additional pathologies and with the greater omentum positioned almost entirely in the upper abdomen (Figure 1(a)). Intravenous antibiotic therapy was started for diagnosis of pneumogenic sepsis.

After six days of hospitalization the patient developed mild abdominal symptoms and his haemoglobin level decreased from 13.5 to 9.9 g/dl while INR increased from 1.41 to 1.51. An abdominal CT showed a moderate sized haemoperitoneum, particularly in the upper abdomen, left anterior perihepatic space, and surrounding a significantly enlarged segment of an omental artery in the left upper abdomen. Spots of enhanced tiny vessels also were visible in the right upper abdomen ventral to the liver (Figure 1(b)). SAM of the left omental artery (LOA) was suspected. As the patient remained haemodynamically stable with a borderline coagulopathic status, a noninterventional therapeutic approach was initially agreed upon. Fresh frozen plasma and erythrocyte concentrates were administered. Despite this therapy the haemoglobin levels further decreased to 7.8 g/dl during the next three days, so that an abdominal control CT was performed. This demonstrated slight progression of the haemoperitoneum. After multidisciplinary discussion the radiological department was asked to perform catheter angiography, if possible with transcatheter embolization.

**Figure 2:**
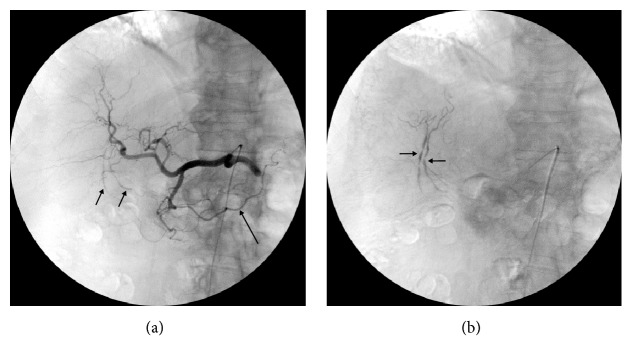
Digital subtraction angiograms. Common hepatic arteriogram (a) with the right gastroepiploic artery forming the omental arch (arrow) and two small right omental branches with multisegmental dilatation (small arrows), better visible in the late phase (b).

**Figure 3:**
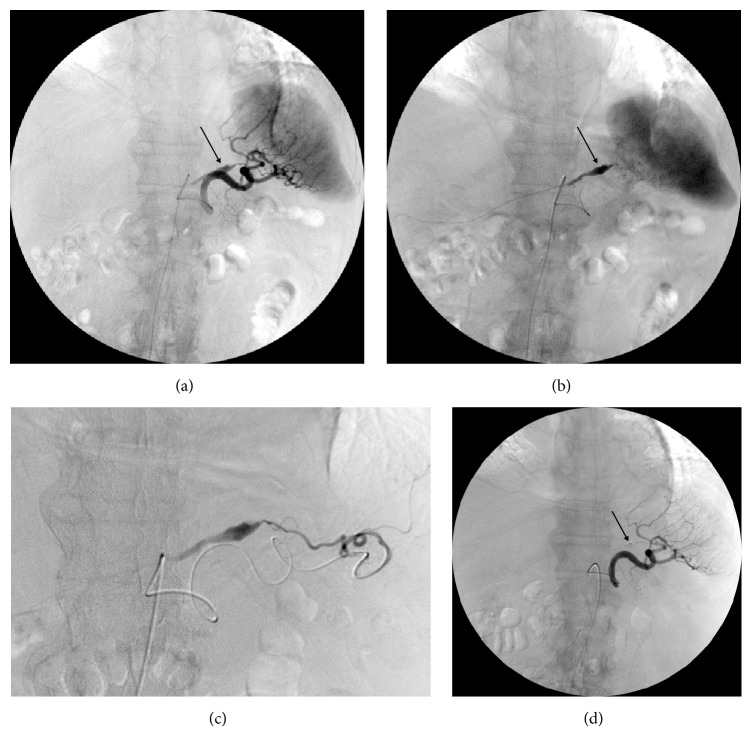
Digital subtraction angiograms. Splenic arteriogram (a) with a markedly dilatated segment, respectively, spindle-shaped aneurysm (arrow) of the left omental artery (LOA), better visible in the late phase (b) and with separate origin of the LOA from an inferior splenic pole artery. Superselective arteriogram of the LOA (c) before embolization (noteworthy, the tortuous microcatheter in the substantially elongated splenic artery). Postembolization arteriogram of the splenic artery demonstrating NBCA-casting and stasis of the LOA (arrow) with preserved splenic arteries (d).

Digital subtraction angiography (DSA) with selective superior mesenteric, common hepatic and splenic arteriogram was performed. These revealed an anatomic variant with absence of the left gastroepiploic artery (left gastroomental artery), the gastroepiploic arch being exclusively formed by the right gastroepiploic artery (right gastroomental artery), and consecutively a separate origin of the LOA (left omental branch) from an inferior pole splenic artery (Figures 2 and 3). Additionally, two tiny omental branches of the right gastroepiploic artery with small multisegmental dilatation and a typical “wind-sock” formed aneurysm of the LOA were visible (Figures 2 and 3). Although no active bleeding was detected, the aneurysm of the LOA was considered to be the obvious cause for the haemoperitoneum.

Superselective catheterization of the LOA through splenic artery and lower pole splenic artery with a microcatheter (2.7-F Progreat, Terumo, Tokyo, Japan) was technically challenging due to vessel tortuosity but succeeded, however, only just a few centimeters proximal to the aneurysm. The decision was made to embolize the LOA with N-butyl-2-cyanoacrylate (NBCA). Approximately 1 ml of a 1:3 mixture of NBCA (Histoacryl; B. Braun, Melsungen, Germany) with iodized oil (Lipiodol ultrafluid; Guerbet, Villepinte, France) was injected in the usual manner. The final DSA control confirmed the complete embolization of the aneurysm of the LOA with preservation of the splenic vessels (Figure 3).

Postinterventional course was uneventful with no signs of omental infarction and with increase of haemoglobin levels up to normal levels.

One month after embolization and after therapy of his protracted pneumonia the patient was transferred to another hospital for early rehabilitation in a satisfactory general condition.

## 3. Discussion

Omental haemorrhage with or without haemoperitoneum is caused by trauma, aneurysm, vasculitis, neoplasm, torsion of the greater omentum, peritonitis, anticoagulant therapy or coagulopathy, and omental pregnancy or with no recognizable cause is referred to as idiopathic. The condition will generally be treated conservatively, in particular by correction of coagulopathy or surgically by means of emergency operation.

With regard to splanchnic aneurysms, the omental artery aneurysm is considered to be the most uncommon [6]. Omental artery aneurysms usually are recognized only after aneurysm rupture and surgery [7, 8].

Radiological literature covering the topic “embolization of omental arteries” is mostly exclusively limited on chemoembolization of hepatocellular carcinomas with additional blood supply from parasitized omental arteries, in which case the corresponding omental artery often is hypertrophied and easier to be catheterized than in healthy subjects [9–11]. In a complete different context of tumour disease Parmar et al. described a case of embolization of omental branches for treatment of disseminated peritoneal leiomyomatosis [12].

Apart from this, there are very limited reports of transcatheter embolization in the event of omental bleeding. We were able to find only four documented cases, most of them relating to “idiopathic” or “spontaneous” omental bleeding without suspected SAM [13–16]. Of these, at least two case descriptions are of embolization of the left gastroepiploic artery and not an omental artery [14, 15]. Takahashi embolized the left gastroepiploic artery, because the culprit vessel of the omentum was too thin to be selected, as did Matsumoto. Only the publication of Yasuoka et al. describes a case of haemoperitoneum due to SAM with embolization of “the greater omental artery branched off from the intrasplenic artery,” that is to say, a similar case to ours; however embolization “did not realize hemostasis” and patient was operated on with partial resection of the greater omentum the same day [13].

In addition we found one case report with haemoperitoneum “as a result of segmental mediolytic arteritis of an omental artery” diagnosed after primary surgery with omental segment resection [17].

In this respect, our case report is the first one of embolization of an omental artery for bleeding due to SAM with both technical and clinical success.

One reason for this might be that omental branches usually are small and branch at an acute angle from the gastroepiploic artery [18] and so superselective catheterization of them might be technically challenging and not always be feasible. Even in cases of hepatocellular carcinomas with additional blood supply from parasitized omental arteries these can be catheterized only in 64-73%, depending on the calibre of the used microcatheter [10, 19].

Blood supply of the omentum according to standard anatomical textbooks is provided by the right and left gastroepiploic artery, both deriving from the celiac trunk. However, anatomical and radiological studies demonstrate a wide range of varieties in this respect and the impossibility of predicting any standard pattern of vascularization [20].

The anatomical variant of our patient with absence of the left gastroepiploic artery and an omental branch directly from the splenic artery has been described in the radiological literature [9, 21] and corresponds to a type 5 of the omental vascular arcade as described by Alday and Goldsmith in their classic paper [22], where “the terminal branch of the splenic artery is not part of the gastroepiploic arch but instead connects directly into the left omental artery.”

In our case with the LOA as a direct branch of the splenic artery on one hand this could have made superselective catheterization of the vessel easier, although the pronounced tortuosity of the splenic artery on the other hand was not a favourable prerequisite for this at all.

## 4. Conclusion

In cases of spontaneous haemoperitoneum SAM should be considered as a cause. The diagnosis can be confirmed and the culprit artery be identified with CT. In the event of an omental artery being the culprit vessel and under the provision of a hemodynamically stable patient transcatheter embolization of the omental branch or, if this is not feasible, of the corresponding gastroepiploic artery should be considered at least as a valuable alternative to a surgical procedure.
